# Applying Data Mining to Investigate Cancer Risk in Patients with Pyogenic Liver Abscess

**DOI:** 10.3390/healthcare8020141

**Published:** 2020-05-22

**Authors:** Jau-Shin Hon, Zhi-Yuan Shi, Chen-Yang Cheng, Zong-You Li

**Affiliations:** 1 Department of Industrial Engineering & Enterprise Information, Tunghai University, Taichung 407, Taiwan; honjs@thu.edu.tw (J.-S.H.); zyshi@vghtc.gov.tw (Z.-Y.S.); wer3329@gmail.com (Z.-Y.L.); 2 Infectious Control Center, Taichung Veterans General Hospital, Taichung 407, Taiwan; 3 School of Medicine, National Yang-Ming University, Taipei 106, Taiwan; 4 Department of Industrial Engineering & Management, National Taipei University of Technology, Taipei 106, Taiwan

**Keywords:** cancer risk, pyogenic liver abscess, hepatocellular carcinoma, colon cancer, data mining, decision tree

## Abstract

Pyogenic liver abscess is usually a complication of biliary tract disease. Taiwan features among the countries with the highest incidence of colorectal cancer (CRC) and hepatocellular carcinoma (HCC). Few studies have investigated whether patients with pyogenic liver abscess (PLA) have higher incidence rates of CRC and HCC. However, these findings have been inconclusive. The risks of CRC and HCC in patients with PLA and the factors contributing to cancer development were assessed in these patients. The clinical tests significantly associated with cancers in these patients with PLA were determined to assist in the early diagnosis of these cancers. Odds ratios (ORs) and 95% confidence intervals (CIs) were determined using binary logistic regression Cancer classification models were constructed using the decision tree algorithm C5.0 to compare the accuracy among different models with those risk factors of cancers and then determine the optimal model. Thereafter, the rules were summarized using the decisi8on tree model to assist in the diagnosis. The results indicated that CRC and HCC (OR, 3.751; 95% CI, 1.149–12.253) and CRC (OR, 6.838; 95% CI, 2.679–17.455) risks were higher in patients with PLA than those without PLA. The decision tree analysis demonstrated that the model with the PLA variable had the highest accuracy, and that classification could be conducted using fewer factors, indicating that PLA is critical in HCC and CRC. Two rules were determined for assisting in the diagnosis of CRC and HCC using the decision tree model.

## 1. Introduction

Biliary tract disease is the most common cause of liver abscess. There has been an increasing incidence of PLA arising from malignancies and their treatment, including liver metastasis, and complications of transarterial chemoembolization or radiofrequency ablation [1]. The annual incidence of pyogenic liver abscess for all age groups increased gradually in Taiwan from 10.83 per 100,000 person-years in 2000 to 15.45 per 100,000 person-years in 2011 [2]. Pyogenic liver abscess occurred more commonly in patients of the male sex, of older age (>50 years), and with lower family income [2]. The annual incidence rates of PLA are much lower in Western countries, for example, 2.3 per 100,000 population in Canada [3] and 1.0 per 100,000 population in Denmark [4]. Moreover, Kao et al. (2012) indicated that patients with pyogenic liver abscess (PLA) are 3.83-, 7.87-, 34-, and 5-times more likely to develop gastrointestinal, liver, biliary, and colon cancers, respectively [5].

According to the data published by the Statistics Department of Taiwan Health and Welfare Ministry, the top 10 causes of death in Taiwan in the year of 2017 were chronic diseases. Of these, malignant tumors represented the main cause of death in Taiwan, followed by heart disease, cerebrovascular diseases, diabetes, and pneumonia. Malignant tumors accounted for 28% of the total mortality [6].

Only few population-based studies have reported increased incidence of GI tract caner in patients with PLA [7,8]. Whether patients with pyogenic liver abscess (PLA) are relatively more likely to develop gastrointestinal cancer needs to be clarified. In addition, these liver cancer and colorectal cancer (CRC) are difficult to detect early. Consequently, the development of more effective methods of early detection is required. 

Therefore, the primary purpose of this study was to analyze the risks of CRC and HCC in patients with PLA, the factors contributing to cancer development in these patients, and the clinical tests for the diagnosis of these cancers. The medical records and clinical data were retrieved from the database. Statistics and data mining technology were used to identify potential risk factors associated with these cancers. The optimal models of decision tree analysis were determined to help the clinicians identify the appropriate clinical tests for the early diagnosis of these cancers and increase the healthy lifespan of patients.

## 2. Related Studies

### 2.1. Liver Cancer

Liver cancer is reported to be the sixth most common cancer and the fourth leading cause of cancer death worldwide in 2018, with about 841,000 new cases and 782,000 deaths annually [9]. The highest annual incidence rates are observed mainly in less developed regions of the world, including several countries in Northern and Western Africa (Egypt, the Gambia, Guinea), and Eastern and South-Eastern Asia (Mongolia, Cambodia, and Vietnam). The age-standardized incidence rates per 100,000 of liver cancers in South-Eastern Asia was 20.1 for males and 6.6 for females in 2018. Taiwan features among the countries with highest incidence rates of liver cancer. The age-standardized incidence rates per 100,000 of liver cancers in Taiwan was 46.5 for males and 18.2 for females in 2015 [6]. Lower annual incidence rates of liver cancers are observed in developed countries in Southern Europe, North America, and Northern Europe. The age-standardized incidence rates per 100,000 of liver cancers in North America was 10.1 for males and 3.4 for females in 2018 [9].

Primary liver cancer includes hepatocellular carcinoma (HCC) (comprising 75–85% of cases), intrahepatic cholangiocarcinoma (comprising 10–15% of cases), and metastatic cancers from other organs. The main risk factors for HCC are chronic infection with hepatitis B virus (HBV) or hepatitis C virus (HCV), heavy alcohol intake, obesity, type 2 diabetes, and aflatoxin-contaminated foodstuffs [9].

Patients with HCC have cirrhosis resulting from the following one or more etiologic risk factors, such as HCV infection, HBV infection, and heavy alcohol drinking. HCC develops at an annual incidence of 2–7% in patients with active viral (HCV or HBV) hepatitis with liver cirrhosis in the United Sates. Because of the advent of highly effective and well-tolerated direct-acting antiviral agents for the treatment of HCV infection, the number of patients cured of HCV has increased rapidly. With the increasing trend of obesity and metabolic syndrome, NAFLD is now the fastest growing cause of chronic liver disease, cirrhosis, and HCC [10]. 

Based on a recent systematic review of the available data, the authors analyzed 47 studies with 15,158 patients, of whom 6284 (41.4%) had HCC detected by surveillance [10]. HCC surveillance was associated with improved early-stage detection (odds ratio (OR) 2.08, 95% CI 1.80–2.37) and curative treatment rates (OR 2.24, 95% CI 1.99–2.52) [10,11]. HCC surveillance was also associated with significantly prolonged survival (OR 1.90, 95% CI 1.67–2.17) [10]. Because most of the evidence-based studies showed a benefit of the combination of ultrasonography and alpha-fetoprotein (AFP) in improving overall survival, the current American Association for the Study of Liver Diseases Guideline recommends surveillance for patients with cirrhosis by liver ultrasonography with or without AFP every 6 months [12].

The liver enzymes tests, AFP, hepatitis markers, and images studies (including reports of abdominal ultrasonography, computed tomography, and magnetic resonance imaging) were analyzed in this study.

### 2.2. Colorectal Cancer

Colorectal cancer usually originates from a noncancerous growth called a polyp that occurs on the inner lining of the colon or rectum and grows slowly over a period of 10 to 20 years. An adenomatous polyp (or called adenoma) is the most common type. Although all adenomas have the potential to become cancerous, only fewer than 10% are estimated to turn into cancer. Cancer arising from the inner lining of the colorectum is called adenocarcinoma and accounts for approximately 96% of all CRCs [13].

The incidence rates of CRC are three-to-four-times more common in developed than in developing countries and more common among men than women. The age-standardized incidence rate per 100,000 of CRC in both sexes is 19.7 in the world (the statistics are 23.6 for males and 16.3 for females). The age-standardized incidence rate among men is about 30.1/100,000 in high-HDI (human development index) nations, while it is 8.4 in low-HDI nations; and the same statistics for women are 20.9 and 5.9, respectively [14]. Developed countries are at the highest risk of colon and rectal cancer. For colon cancer, Southern Europe, Northern Europe, and Australia/New Zealand are the regions with highest incidence rates. For rectal cancer, the highest regions are Eastern Europe, Australia/New Zealand, and Eastern Asia. North America also is among the countries with the highest incidence rates for both cancers. In contrast, Africa, as well as Southern Asia, have the lowest incidence rates for both cancers in both sexes [14]. However, the incidence rates of colon cancer in Taiwan is higher than other Southern Asian countries. The age-standardized incidence rates per 100,000 of colon cancers in Taiwan was 52.1 for males and 34.9 for females in 2015 [6].

In the United States, CRC is the third most commonly diagnosed cancer and the second leading cause of death from cancer [15]. About 39% of patients with CRC are diagnosed with localized-stage disease, 35% are diagnosed with regional-stage disease, and 21% are diagnosed with metastatic-stage disease. The five-year survival of CRC is 90% if it is diagnosed while still localized (i.e., confined to the wall of the bowel), but only 71% for regional disease (i.e., disease with lymph node involvement), and only 14% if distant metastasis is present [15].

Screening for colorectal cancer is recommended by American Society of Clinical Oncology. For people who are asymptomatic, aged 50 to 75 years, without family history of colorectal cancer, at average risk, and in settings with high incidences of colorectal cancer, the following screening options are recommended: Guaiac fecal occult blood test and fecal immunochemical testing (basic), flexible sigmoidoscopy (add option in limited setting), and colonoscopy (add option in enhanced setting) [16]. The data of these three tests were analyzed in this study.

### 2.3. Research on Data Mining in Disease Prediction

Hui and Jha (2000) proposed that new technologies can aid in understanding the relationship between data and in clustering large amounts of stored data [17]. This type of knowledge acquisition from data, which are the type of data contained in a database, data warehouse, and abnormal part of the data, is called data mining. Data mining is used to determine the relationships between data from a large amount of data stored in a database and to extract predictable information to aid in decision-making. Therefore, a method of data mining was used in this study to systematically analyze patient examination data and to determine methods to aid in disease prediction and diagnosis.

Han, Pei, and Kamber (2011) proposed a seven-step data mining process: (1) Cleaning data to remove noise; (2) integrating data from different sources; (3) selecting data related to the target from databases; (4) transforming data by simplifying, converting, or encoding them; (5) applying data mining techniques to extract useful knowledge; (6) evaluating patterns using metrics to identify useful information; and (7) presenting the results visually to aid decision-making [18].

Luk et al. (2007) investigated data on liver tissue proteomes and proposed an artificial neural network and decision tree model analysis of liver cancer proteomes. They concluded that classification-like neural networks were superior to decision trees, but decision trees were more appropriate for the discovery of markers than neural networks [19].

Kurosaki et al. (2012) proposed a data mining model that used simple and readily available factors that could aid in identifying patients at a high risk of HCC among patients with chronic hepatitis C using a decision tree and logistic regression [20]. The collected data were age, gender, and blood test reports. They concluded that decision trees were easy to use and suitable for determining the patients at high risk. Moreover, patients with hepatitis C liver on Pegylated Interferon (PEG-IFN) had a lower risk of cancer.

A considerable proportion of domestic and foreign research has focused on neural networks, decision trees, and logistic regression. These three methods have advantages and limitations. However, the majority of scholars agree that decision tree extraction rules are more suitable for identifying rules to assist in diagnosis. Therefore, decision trees were used for data mining in the present study. The conclusions obtained by the decision tree can be explained, which facilitates identifying the causal association rules between the input and cancer, and the decision tree is thus more suitable for this study than the other methods.

## 3. Methodology

**Figure 1:**
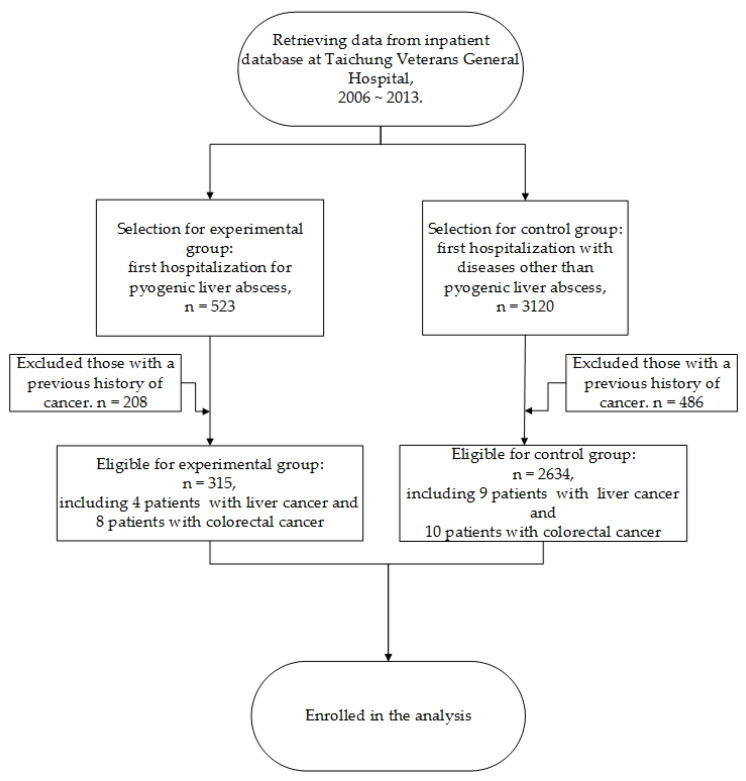
Selection procedure of eligible patients for the analysis.

Patient data provided by the Clinical Informatics Research and Development Center, Taichung Veterans General Hospital, Taiwan, were analyzed. The data acquisition period was from January 1, 2006, to December 31, 2013. Research participants were divided into two groups: Experimental and control groups. The experimental group was comprised inpatients with a PLA with a primary diagnosis ICD-9-CM code 572.0. Patient data, including age and gender, were tracked from the date of first hospitalization or first diagnosis of liver abscess and were followed up for 10 years. The control group was selected based on pairing with the experimental group according to age and genders. The ratio of experimental to control participants was 1:10. All patients hospitalized from 2006 to 2013 were sampled by random sampling. Their clinical data were tracked from one year before the date of the first hospitalization and were followed up thereafter until 2013. Patients with AIDS (ICD-9-CM code 043) or a history of cancer before the first hospitalization were excluded from both groups. The flowchart of patient selection and exclusion in this study is illustrated in Figure 1.

Research on the collected data items was divided into two parts. The first part was general basic information (patient age and gender). The second part was the diagnosis data obtained in the hospital using the ICD-9-CM codes: Diabetes (ICD-9-CM code 250.0), hepatitis B (ICD-9-CM codes 070.2 and 070.3), hepatitis C (ICD-9-CM codes 070.41, 070.44, 070.51, and 070.54), alcoholic cirrhosis (ICD-9-CM codes 571–571.3), hepatic cirrhosis (ICD-9-CM codes 571.5 and 571.6), fatty liver (ICD-9-CM code 571.8), abnormal liver function (ICD-9-CM code 794.8), obesity (ICD-9-CM code 278.0), gastric cancer (ICD-9-CM code 151), CRC (ICD-9-CM codes 153 and 154), liver cancer (ICD-9-CM codes 155.0 and 155.1), biliary tract cancer (ICD-9-CM code 156), pancreatic cancer (ICD-9-CM code 157), breast cancer (ICD-9-CM codes 174 and 175), nasopharyngeal carcinoma (ICD-9-CM code 147), lung cancer (ICD-9-CM code 162), cervical cancer (ICD-9-CM code 180), and kidney cancer (ICD-9-CM code 189). The following laboratory test data were obtained: the tumor indexes CEA, CA-199, and AFP; blood characteristics, comprising blood glucose, AST, ALT, bilirubin, albumin, total cholesterol, platelet, hemoglobin, and triglyceride levels; anti-HCV antibody, HBs Ag, HBe Ag, anti-HBs antibody, and anti-HBc antibody levels; and fecal occult blood examination results. Colonoscopy results were also documented. Imaging examinations, including computed tomography, abdominal ultrasound, and magnetic resonance imaging reports, were also collected. The pathology report included pathological test results.

After data preprocessing and research variable conversion, the data revealed that the inspection items created by each patient were not the same, resulting in the lack of some information. Originally, this problem was to be addressed by treating the differences as missing values or by taking the average value of the data for treatment. After a discussion with the hospital’s physicians, it was considered that if a patient had not undergone a test, it indicated that the patient did not need the test. The patient was in the healthy group and was categorized as 0.

The descriptions of patients’ situations in colonoscopy and imaging examination reports also differed based on physicians’ habits of writing. Reports displayed different writing methods but similar diagnoses. For instance, similar statements appeared on liver ultrasound reports, such as a 3.5-cm abscess in the left liver lobe, 3.7-cm abscess in the right liver lobe, or 4.2-cm the left liver abscess. Each diagnosis was categorized as “hepatic abscess” to reduce excessive variables. Therefore, the data were converted into scores according to the standard value of each test item: not checked → 0, check result was normal → 1, or check result was abnormal → 2.

### 3.1. Decision Tree

The decision tree is the original classification algorithm of Iterative Dichotomiser 3 (ID3), the predecessor of C4.5 and C5.0, proposed by Quinlan and other scholars in 1986 [21]. The algorithm uses information gains as the criterion for segmentation. Information gain is described as follows. First, the information theory is that if there are *K* kinds of results in one event and the corresponding probability is *P_i_*, then the amount of information obtained after the event occurs is given as

(1) $$I=-\left(P_{1}\times \log_{2}\left(P_{1}\right)+P_{2}\times \log_{2}\left(P_{2}\right)+\cdots +P_{k}\times \log_{2}\left(P_{k}\right)\right)$$

Second, information gain is classified into two categories (with and without cancer), and *X* is the predictive variable (category *k*), where n is the total number of samples ($m_{i1}$ is the total number of carcinoma markers in the sample). After classification based on the *X* variable, *m_i_* is obtained and is the total number of samples in class *X* = *i* ($m_{i1}$ is the number of carcinoma markers in class *X* = *i*). According to the variable *X*, the information gains of *n* samples divided into *m_1_*, *m_2_*, …, *m_k_* are as follows:

(2) $$\mathrm{Gain}\left(X\right)=I\left(n,n_{1}\right)-E\left(X\right)$$

(3) $$I=-\left(\left(\frac{n_{1}}{n}\right)\times \log_{2}\left(\frac{n_{1}}{n}\right)+\left(1-\frac{n_{1}}{n}\right)\times \log_{2}\left(1-\frac{n_{1}}{n}\right)\right)$$

(4) $$E\left(x\right)=\left(\frac{m_{1}}{n}\right)\times I\left(m_{1},m_{11}\right)+\left(\frac{m_{2}}{n}\right)\times I\left(m_{2},m_{21}\right)+\cdots +\left(\frac{m_{k}}{n}\right)\times I\left(m_{k},m_{k1}\right)$$

When building a decision tree, the goal of each node is to segment the training data to identify the optimal segmentation and segmentation points. Therefore, for each node generated, the training subset is removed to calculate the quantity of information gain, and the value with the largest information gain is regarded as the branch node. The next branch node is then selected according to the recursive action until each training material belongs to a category. The decision tree algorithm C5.0 used here was improved by Quinlan’s C4.5 algorithm proposed in 1993 [22]. Boosting was used to improve the model and enable ID3 to manage continuous attributes. The software operation speed was improved, and the memory resources were less occupied.

### 3.2. Evaluation Model

A confusion matrix is used to evaluate the performance of a classification model on a set of test data for which the true values are known. When the classification problem has two categories, the confusion matrix obtained is as listed in Table 1. The upper left and lower right corners of the matrix indicate correct prediction, and the upper right and lower left corners are the incorrect predictions.

Evaluation of the correctness of the classification model in the supervised learning algorithm and the correct rate of future predictions can reveal the degree of confidence in the classification in the prediction model and can be used to select specific classifications.

The evaluation indicators used in the classification model employed in this study had the following three points. First,

(5) $$\mathrm{Forward}\;\mathrm{rate}=\frac{TP}{TP+FN}$$

in which the correct forward classification number is divided by the total positive classification number, which gives the sensitivity (true-positive rate). Second,

(6) $$\mathrm{Correct}\;\mathrm{rate}=\frac{TN}{TN+FP}$$

in which the correct negative classification number is divided by the total negative classification number, which gives the specificity (true-negative rate). Third,

(7) $$\mathrm{Correct}\;\mathrm{rate}=\frac{TP+TN}{TP+TN+FP+FN}$$

in which the correct number of classifications is divided by the total number of examples, which gives the overall classification accuracy rate. The similarity ratio algorithm for assessing the clinical significance of the similarity ratio values is as follows (Table 2).

LR (+) = true-positive rate/false-positive rate = Sensitivity/(1 − Specificity)(8)

LR (−) = false-negative rate/true-negative rate = (1 − Sensitivity)/Specificity(9)

## 4. Results

### 4.1. Statistical Results

All statistical analyses were performed using SPSS (version 20.0, IBM, Armonk, NY, USA). According to the Ministry of Health and Welfare statistics, cancer incidence is affected by age and gender. Therefore, the odds ratios (ORs) for cancer in the experimental group (with liver abscess) should be calculated according to age and gender. The intergroup differences in the effects of age and gender were determined using *t* and chi-square tests, respectively. After confirming the absence of a significant difference in age and gender between the experimental and control groups, the incidence of CRC and liver cancer was analyzed. The ORs were calculated using logistic regression, and CRC and liver cancer risks were analyzed. These results are listed in Table 3 and Table 4.

As shown in Table 5 and Table 6, significant differences were observed in the risks of CRC and liver cancer between the experimental group (with PLA) and the control group (without PLA). The OR for CRC in the experimental group was 6.838 (95% CI, 2.679–17.455). Thus, CRC risk in the experimental group was 6.838-times the risk in the control group. The OR for liver cancer in the experimental group was 3.751 (95% CI, 1.149–12.253). Thus, the liver cancer risk in the experimental group was 3.751-times the risk in the control group.

### 4.2. Decision Tree Analysis Results

We demonstrated that the ORs for cancer in the experimental group were higher than those in the control group. Therefore, the risks of CRC and liver cancer in patients with PLA were higher than those in the average person. Thus, PLA was used as a research variable in a predictive model, which was compared to the prediction model with PLA as an established prediction model. We used the C5.0 decision tree method in SPSS to extract classification rules from the data.

**Figure 2:**
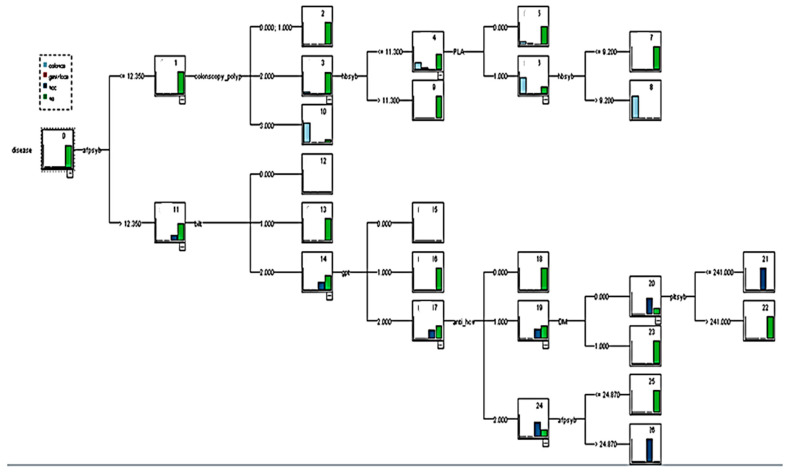
Decision tree model 1 (with pyogenic liver abscess variable).

First, the PLA variable was added to establish a prediction model, which was named decision tree model 1 (with PLA). The overall tree diagram of this model is illustrated in Figure 2. After 10-fold cross-validation, the model demonstrated an overall classification accuracy rate of 98.8%, and numerous classification rules could be compiled from the overall tree diagram (Table 7 and Table 8).

The explanation was based on Rule 1 of CRC. When the AFP check value was 12.3 (ng/mL) and the colonoscopy polyp = 3 (an adenoma is identified using colonoscopy), the decision tree identified eight people, which reflected the confidence of the rule. The standard was 87.5%.

**Figure 3:**
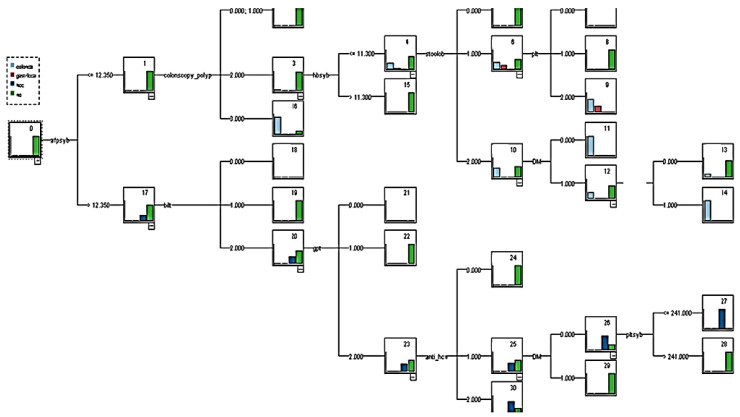
Decision tree model 1 (no pyogenic liver abscess variable).

The PLA variable was then removed and was used in the established prediction model. The model was named decision tree model 1 (without PLA), and the overall tree diagram of this model is illustrated in Figure 3. After 10-fold cross-validation, the model revealed that the overall classification accuracy rate was 98.5%. Classification rules were compiled from the overall tree diagram (Table 9 and Table 10).

The models model 1 (with PLA) and model 1 (without PLA) demonstrated that the classification rules for liver cancer were almost identical to the classification rules for CRC. However, these models exhibited a considerable difference, which indicated a relationship between PLA and CRC. Therefore, this study also established a predictive model for CRC and compared the addition of the variable related to bacterial liver abscess to understand whether bacterial liver abscess would increase the accuracy of classification.

**Figure 4:**
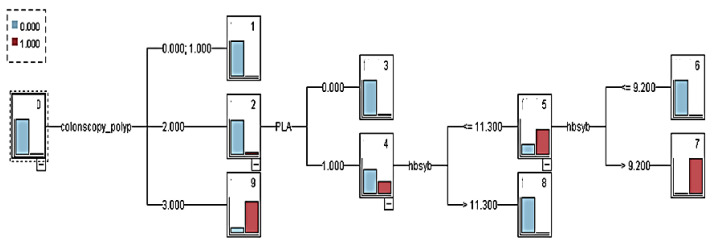
Decision tree model 2 (pyogenic liver abscess variable).

The PLA variable was included in the prediction model of CRC. The model was named decision tree model 2 (with PLA). The overall tree diagram of this model is illustrated in Figure 4. After 10-fold cross-validation, the model displayed an overall classification accuracy rate of 99.5% and revealed classification rules that could be compiled from the overall tree diagram (Table 11).

**Figure 5:**
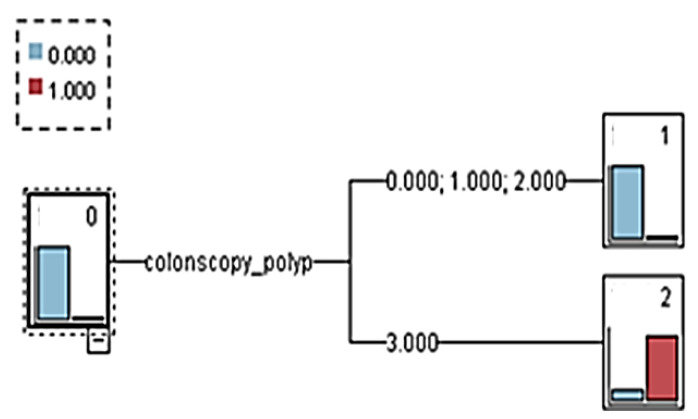
Decision tree model 2 (no pyogenic liver abscess variable).

A CRC prediction model was established without the PLA variable, named decision tree model 2 (without PLA). The overall tree diagram of this model is illustrated in Figure 5. After 10-fold cross-validation, the model displayed an overall classification accuracy rate of 99.5%, and its classification rule could be seen as “rule 1: if the colonoscopy polyp = 3 (adenomas), there is a large intestine Cancer (8, 0.875).”

### 4.3. Decision Tree Model Evaluation

Two approaches were used to determine the optimal predictive model. The correct rate and the application surface were determined based on the sensitivity, specificity, and overall classification accuracy of the 10-fold cross-validation, and whether it accorded with the current situation and had a reference value was assessed.

The classification rule of the CRC model of model 2 (without PLA variants) was too simple because it judged using only one factor. This classification rule categorized polyps in colonoscopy as having no CRC. However, Garborg et al. (2013) reported that adenomas may not be necessary for the development of CRC [24]. Furthermore, physicians can only distinguish hyperplastic polyps or adenomatous polyps during colonoscopy. Therefore, polyps can still be adenomatous polyps after pathological confirmation. Consequently, the CRC model was excluded as the optimal predictive model (model 2, without the PLA variable). The model of CRC summarized in model 2 (with PLA variants) was the same as the CRC rule in model 1 (with PLA variant). Therefore, no comparison was made. To ensure that the established model had certain accuracy and feasibility, we determined whether it had clinical significance by calculating Likelihood Ratios (LR). The similarities between the four models were calculated (Table 12).

Table 12 indicates that all four models belonged to effective classification rules. Therefore, they could be used to assist in the diagnosis with certain accuracy. Therefore, the sensitivity, specificity, and overall classification accuracy of the 10-fold cross-validation were further compared to determine the optimal model. The sensitivity, specificity, and overall classification accuracy of model 1 after cross-validation are summarized in Table 13.

From the sensitivity and specificity of the model and the overall prediction accuracy, we determined that the accuracy of the two models were similar. After discussing our findings with a doctor involved in this study, it was determined that the clinical situation could be diagnosed using fewer factors. Implementing these changes would improve the predictive power of the model. Therefore, the model with PLA in decision tree model 1 was the optimal model presented in this study.

### 4.4. Discussion

The main risk factors for HCC include chronic hepatitis caused by HBV or HCV, alcoholic liver disease, and nonalcoholic liver disease (NAFLD) [25,26]. Several population-based studies conducted in various geographic areas have reported a significantly increased incidence of HCC in patients with diabetes and obesity [27,28]. Obesity, type 2 diabetes mellitus (T2DM), and dyslipidemia are the most common metabolic risk factors associated with NAFLD [26,29]. 

NAFLD is mainly a consequence of excess caloric intake and lack of physical activity [30]. A low calorie diet rich in protein is associated with improved lipid profile, glucose homeostasis, and liver enzymes in NAFLD, and decreases in body mass index (or body fat mass) [31]. With the high levels of monounsaturated fatty acids, extra virgin olive oil (EVOO) has the relevant molecular effects involving in the prevention or resolution of liver damage by inducing the cellular antioxidant response, preventing the cellular inflammatory response, and preventing endoplasmic reticulum stress and lipogenic response [32]. 

Since the prevalence of obesity and its related comorbidities has increased globally in recent decades, NAFLD has become progressively more common. Currently, there is no effective drug therapy for NAFLD [30]. Therefore, interventions in lifestyles and diet control remain the primary approach [30]. However, the information of lifestyle interventions (such as reduction of body weight, increasing physical activity, regular exercise program), dietary interventions (such as low calorie and rich protein diet, Mediterranean diet rich in olive oil), and amino acid supplementation interventions were not recorded in the medical records. Therefore, they were not included in this study.

## 5. Conclusions

This study revealed that patients with PLA had higher rates of liver cancer and CRC than other hospitalized patients. The OR for CRC was 6.838 (95% CI, 2.679–17.455), and the OR for liver cancer was 3.751 (95% CI, 1.149–12.253). 

CRC and liver cancer are chronic diseases, with few symptoms during their early stages. Therefore, most colorectal and liver cancers are discovered in the middle and late stages. However, when these cancers are discovered in the early stages, survival rates are very high. This study aimed to establish preventive interventions by establishing guidelines for prevention and early detection. In the decision tree used to identify the optimal model, two rules for CRC and liver cancers were identified. The classification rules for CRC could be classified with fewer factors and had greater prediction accuracy than the classification rule without the addition of PLA. Therefore, the potential risk factor for liver abscess is key in identifying high-risk individuals for CRC.

A rule among CRC in patients with PLA was identified from the two most accurate CRC rules, which could be used by physicians to assist in the early diagnosis of CRC for patients who present the following characteristics: AFP < 12.3 ng/mL, polyps in colonoscopy, heme = 9.2–11.3 g/dL, and presence of PLA. The tumor index AFP is principally used for hepatocarcinoma testing, and the pattern of model 2 (with PLA variants) suggested that this indicator is unnecessary for CRC prediction. Therefore, when caring for patients with PLA, clinicians should pay particular attention to hemoglobins and the presence of a large intestine polyp.

The relationship between PLA and CRC or liver cancer has scarcely been discussed in the literature. However, this relationship warrants further exploration. This study is limited by research limitations and gaps in the research process. Therefore, we provided suggestions for subsequent research. First, the data enrolled 315 PLA cases and 2634 control cases including eight CRC and four liver cancer cases with a history of PLA and ten CRC and nine liver cancer cases without a history of PLA. The sample size was still insufficient. If more cases were obtained, the diagnostic models presented could be assessed with less variability and bias. Second, data were collected from medical records and databases. Therefore, certain risk factors for cancer, such as living habits, genetic factors, and alcohol abuse, could not be identified in the clinical database. With these factors, the analysis would lead to a more comprehensive model. Third, the research variables were factors related to CRC and liver cancer and known related factors. Assessing general health-related information may reveal factors that have not been identified and provide new reference indicators for medical personnel.

## Figures and Tables

**Table 1:** Confusion matrix of two categories.

Confusion Matrix		Real Category	
		+	−
Forecast category	+	Correct positive number (TP)	Error positive number (FP)
	−	Error negative number (FN)	Correct negative number (TN)

Source: Li Shengqi, Wu Yizhen (translation) (2011).

**Table 2:** Clinical significance of similarity values.

Likelihood Ratios	Interpretation
>10	Strong evidence to rule in disease
5–10	Moderate evidence to rule in disease
2–5	Weak evidence to rule in disease
0.5–2.0	No significant change in the likelihood
0.2–0.5	Weak evidence to rule out disease
0.1–0.2	Moderate evidence to rule out disease
<0.1	Strong evidence to rule out disease

Source: Haynes, et al [23].

**Table 3:** Comparison of demographic characteristics age (t-test).

Age Characteristics	Control Group (*n* = 2634), *n* (%)	Test Group (*n* = 315), *n* (%)	*p*-Value
Ages			0.142
<40	297 (11.3)	30 (9.5)	
40–49	347 (13.2)	39 (12.4)	
50–59	802 (30.4)	95 (30.2)	
60–69	559 (21.2)	69 (21.9)	
>70	629 (23.9)	82 (26.1)	

**Table 4:** Comparison of demographic characteristics gender (chi-square test).

Gender Characteristics	Control Group (*n* = 2634), *n* (%)	Test Group (*n* = 315), *n* (%)	*p*-Value
Gender			0.587
Female	731 (27.8)	92 (29.2)	
Male	1903 (72.2)	223 (70.8)	

**Table 5:** Logistic regression colorectal cancer odds ratio.

Colorectal Cancer Odds Ratio	Pyogenic Liver Abscess (Y)	Pyogenic Liver Abscess (N)	Total	Winning Ratio	95%CI	*p*-Value
Colorectal cancer (Y)	8	10	18	6.838	2.679−17.455	*p* < 0.001
Colorectal cancer (N)	307	2624	2931			
Total	315	2634	2949			

**Table 6:** Logistic regression liver cancer odds ratio.

Liver Cancer Odds Ratio	Pyogenic Liver Abscess (Y)	Pyogenic Liver Abscess (N)	Total	Winning Ratio	95%CI	*p*-Value
Liver cancer (Y)	4	9	13	3.751	1.149−12.253	*p* = 0.0019
Liver cancer (N)	311	2625	2936			
Total	315	2634	2949			

**Table 7:** Model 1 (with bacterial liver abscess) classification results—colorectal cancer.

Rule	AFP (ng/mL)	Colonoscopy Polyps	Hb (g/dL)	Pyogenic Liver Abscess	Inductive Result
1	≤12.3	3			(8, 0.875)
2	≤12.3	2	≤11.3; >9.2	1	(5, 1.0)

**Table 8:** Model 1 (with bacterial liver abscess variable) classification results—liver cancer.

Rule	AFP (ng/mL)	Bilirubin	GPT	Anti-HCV Ab	Diabetes	Platelet (×1000/CUMM)	Inductive Result
1	>12.3; >24.87	2	2	2			(4, 1.0)
2	>12.3	2	2	1	0	<241	(5, 1.0)

**Table 9:** Model 1 (no bacterial liver abscess variable) classification results—colorectal cancer.

Rule	AFP (ng/mL)	Colonoscopy_Polyp	Hb (g/dL)	Fecal Occult Blood	Platelet	Diabetes	Fatty Liver	Inductive Result
1	≤12.3	3						(8, 0.875)
2	≤12.3	2	≤11.3	1	2			(3, 0.67)
3	≤12.3	2	≤11.3	2		0		(2, 1.0)
4	≤12.3	2	≤11.3	2		1	1	(2, 1.0)

**Table 10:** Model 1 (no bacterial liver abscess variable) classification results—liver cancer.

Rule	AFP (ng/mL)	Bilirubin	GPT	Anti-HCVAb	Diabetes	Platelet (×1000/CUMM)	Inductive Result
1	>12.3	2	2	2			(6, 0.67)
2	>12.3	2	2	1	0	≤241	(5, 1.0)

**Table 11:** Model 2 (with bacterial liver abscess) classification results—colorectal cancer.

Rule	Colonoscopy Polyps	Pyogenic Liver Abscess	Hb (g/dL)	Inductive Result
1	3			(8, 0.875)
2	2	1	≤11.3; >9.2	(5, 1.0)

**Table 12:** Likelihood Ratios of the four models.

LR	Model 1 (with PLA) Colorectal Cancer	Model 1 (with PLA) Liver Cancer	Model 1 (No PLA) Colorectal Cancer	Model 1 (No PLA) Liver Cancer
LR(+)	375	Approximate infinite	187.5	750
LR(−)	0.25	0.25	0.25	0.25

**Table 13:** Comparison of Model 1.

Model	Sensitivity	Specificity	10-Fold Cross-Validation
Model 1 (with PLA variant) colorectal cancer	0.75	0.998	98.8%
Model 1 (with PLA variant) liver cancer	0.75	1	
Model 1 (no PLA variable) colorectal cancer	0.75	0.996	98.5%
Model 1 (no PLA variable) liver cancer	0.75	0.999	
